# Microsatellite Alterations Are also Present in the Less Aggressive Types of Adult T-Cell Leukemia-Lymphoma

**DOI:** 10.1371/journal.pntd.0003403

**Published:** 2015-01-15

**Authors:** Marcelo Magalhães, Pedro D. Oliveira, Achiléa L. Bittencourt, Lourdes Farre

**Affiliations:** 1 Laboratory of Experimental Pathology (LAPEX), Gonçalo Moniz Research Center, Oswaldo Cruz Foundation (CPQGM/FIOCRUZ), Salvador, Bahia, Brazil; 2 Department of Dermatology, Complexo Hospitalar Universitário Professor Edgard Santos, Federal University of Bahia, Salvador, Bahia, Brazil; 3 Department of Pathology, Complexo Hospitalar Universitário Professor Edgard Santos, Federal University of Bahia, Salvador, Bahia, Brazil; 4 National Institute of Science and Technology of Tropical Diseases (INCT/DT), Salvador, Brazil; George Mason University, UNITED STATES

## Abstract

**Background:**

Adult T-cell leukemia/lymphoma (ATL) is a mature T-cell neoplasia etiologically linked to HTLV-1. Manifestations of ATL are diverse and different clinical types with different tissue involvement and aggressiveness have been described. The mechanisms that lead to the development of ATL clinical types have not yet been clarified. Considering that in ATL patients HTLV-1 infection generally occurs in childhood, a multistep carcinogenesis model has been proposed. Microsatellite alterations are important genetic events in cancer development and these alterations have been reported in the aggressive types of ATL. Little is known about oncogenesis of the less aggressive types.

**Methodology/Principal Findings:**

In this study we investigated the role of the microsatellite alterations in the pathogenesis mediated by HTLV-1 in the different types of ATL. We examined the presence of microsatellite instability (MSI) and loss of heterozigosity (LOH) in matched pair samples (tumoral and normal) of 24 patients with less aggressive types (smoldering and chronic) and in aggressive types (acute and lymphoma) of ATL. Four microsatellite markers D10S190, D10S191, D1391 and DCC were analyzed. MSI was found in four patients, three smoldering and one chronic, and LOH in four patients, three smoldering and one acute. None of the smoldering patients with microsatellite alterations progressed to aggressive ATL.

**Conclusions/Significance:**

To our knowledge, this is the first report describing the presence of MSI and LOH in the less aggressive types of ATL. These results indicate that microsatellite alterations may participate in the development of the less aggressive types of ATL.

## Author Summary

Adult T cell leukemia/lymphoma (ATL) is a severe neoplasia caused by a retrovirus named human T cell lymphotropic virus type-1 (HTLV-1). There are 5 to 10 million carriers worldwide, but only 2% to 5% will manifest ATL. The patients present different clinical and laboratory features. Therefore, this neoplasia was classified in acute and lymphoma types which presented poor prognosis, with a median survival time around 6 months and the less aggressive chronic and smoldering types. The mechanisms involved in the development of this disease are not well known. Microsatellites are short tandem repeats of DNA spread throughout the genome. Alterations in these sequences have been related to the development of cancers. In Japan, one of the highest endemic areas of HTLV-1 infection, alterations in microsatellites have been observed in the aggressive types of ATL and were related to poor prognosis and progression of disease. In the current study, in which we included many chronic and smoldering patients, we showed that microsatellite alterations were also present in the less aggressive ATL and were not necessarily associated to ATL progression and aggressiveness.

## Introduction

Adult T-cell leukemia/lymphoma (ATL) is a severe mature T-cell neoplasia caused by human T-cell lymphotropic virus type-1 (HTLV-1) [1]. It is estimated that there are approximately 5–10 million HLV-1 carriers worldwide, distributed in endemic areas which include Japan, Caribbean islands, Iran, Central and West Africa and South America [2]. The lifetime risk of developing ATL is estimated to be 2–5% [3],[4]. In Japan, there are about 700 new ATL patients every year [5]. HTLV-1 infection is endemic in the state of Bahia in Northeastern Brazil, where several cases of ATL have already been reported [6]. In Bahia, 14% of the ATL patients also present HTLV-1-associated myelopathy/tropicalspastic paraparesis (HAM/TSP) [6], a chronic inflammatory disease of the central nervous system [7]. ATL is originally classified into four clinical types: acute, chronic, lymphoma and smoldering [8]. The acute and lymphoma types have a poor prognosis, whereas the chronic and smoldering types have a less aggressive clinical course [6]. Because there is a long latency period between infection and the development of ATL, a multistep carcinogenesis model has been suggested for this neoplasia [9]. However, the oncogenic mechanisms implicated in the development of the different clinical types of ATL have yet to be established [10]. Microsatellites are short tandem repeats of DNA with one to six base pairs, which are very polymorphic and interspersed throughout the human genome. Alterations such as microsatellite instability (MSI) and loss of heterozygosity (LOH) have been documented in various tumor types, including hematological malignancies. MSI is associated with the failure of the DNA mismatch repair system [11]. LOH represents somatic allelic deletion in tumor DNA and may be related to inactivation of a tumor suppressor gene. MSI alterations have been reported in the aggressive ATL in Japan [12]–[15]. Hatta *et al.* [12] investigated 22 cases of acute and lymphoma types of ATL and found MSI in 40% of them. These authors suggested that MSI may be involved in the progression of ATL. Hayami *et al.* [13] studied 18 ATL patients, 14 of whom had the acute, three the chronic and one the smoldering type. These authors found MSI in 22% of the cases, all with the acute form of ATL. They suggested that genomic instability may be associated with tumor progression rather than to the development of ATL itself. LOH was reported in two studies including the acute and lymphoma types [14],[15]. The aim of the present study was to investigate the role of MSI and LOH in the pathogenesis mediated by HTLV-1 in patients with different clinical types of ATL.

## Methods

The study included 24 patients with ATL diagnosed at the Professor Edgard Santos Teaching Hospital at the Federal University of Bahia (UFBA), Brazil. Five patients had the acute, nine the chronic, nine the smoldering and one the lymphoma-type [8] (Table 1). Additional samples from patients #10 and #19 were collected after ATL progression from the smoldering to the chronic and from the chronic to the acute phase, respectively. All cases of smoldering ATL were non-leukemic [8], presented only skin lesions and none or less than 4% of atypical cells in peripheral blood smears. Serology for HTLV-1 was performed by enzyme-linked immunosorbent assay (ELISA, Ortho Clinical Diagnostics Inc., USA) and confirmed by Western blot (HTLV Blot 2.4, Genelabs Technologies). All patients were HIV-negative. Eight patients had association with HAM/TSP (Table 1). The diagnosis and classification of ATL and HAM/TSP were based on established criteria [7],[8]. Matched pair samples (tumoral and normal samples from the same patient) were evaluated. DNA was extracted from peripheral blood mononuclear cells (PBMC) using the Cell Culture DNA Mini Kit (Qiagen, Valencia, CA). ) The PBMC were collected prior to the treatment with an association of zidovudine and interferon-α (AZT/INF-α) or chemotherapy, except for patient #11 who has been treated with AZT/INF-α for the last five years. DNA control from each patient was extracted from normal samples (oral mucosa, hair root, nail specimens and/or normal skin or lung tissues included in paraffin blocks), using the QIAamp DNA investigator Kit (Qiagen, Valencia, CA) or QIAamp DNA FFPE tissue kit (Qiagen, Valencia, CA). Microsatellite alterations were investigated using four markers: D10S190 and D10S191 (located at chromosome 10), D11S1391 (located at chromosome 11) and DCC 18S21 (located at chromosome 18). These loci were selected because they are known to present a higher frequency of MSI in aggressive ATL [12]. The primer sequences for PCR amplification of each marker were obtained from the UniSTS database (http://www.ncbi.nlm.nih.gov/unists/). For each marker, the forward primer was fluorescently labeled with FAM, VIC, NED or PET (Applied Biosystems, Foster City, CA). PCR products were combined with formamide and LIZ-500 size standard and analyzed by capillary electrophoresis using an ABI 3100 Genetic Analyzer (Applied Biosystems, Foster City, CA). Data analysis was performed using the Peak Scanner Software (Applied Biosystems, Foster City, CA). MSI was determined by PCR comparing the amplification pattern in the tumoral and normal samples from the same patient. A method based on the intensity of the amplification signal was used to determine LOH [16]. The ratio between the amplification intensity of PBMC samples and that of the normal tissue samples was calculated as previously described [16]. In this analysis, ratio values ≤ 0.6 or > 1.67 were considered representative of LOH. Only patients who were heterozygous for a given locus were considered informative for LOH analysis. The HTLV-1 proviral load was quantified by real-time PCR using 30 ng of DNA and the *TaqMan Fast Universal PCR Master Mix* kit (Applied Biosystems) in the *7500 Fast Real-Time PCR System* (Applied Biosystems). The HTLV-1 tax gene was selected for quantification of viral copies and normalization was carried out using the beta-globin gene.

**Table 1:** Clinical characteristics and results of microsatellite alterations analysis for patients with ATL.

**Case**	**Clinical type**	**Sex**	**Age**	**Lymphocyte count (×10^9^/L)**	**Survival (months)**	**PVL (copies/100 PBMC)**	**Skin lesions**	**Microsatellite alteration**			
								**D10S190**	**D10S191**	**D11S1391**	**DCC**
1	Smoldering	M	9	4.5^£^	23^#^	9.94	Present	-	LOH	NA	MSI
3*	Smoldering	F	17	2.5	48^#^	17.72	Present	-	-	-	-
4	Smoldering	F	76	4	84^#^	16.04	Present	-	-	-	-
5*	Smoldering	F	46	1.8	72^#^	5.57	Present	LOH	-	-	-
6*	Smoldering	F	56	3.1	33	11.34	Present	MSI	-	-	-
7*	Smoldering	M	67	3.7	60^#^	13.17	Present	-	-	-	-
8*	Smoldering	F	52	3.5	120^#^	8.19	Present	-	MSI	-	-
9*	Smoldering	M	72	3.4	108^#^	35.81	Present	-	-	-	-
10^β^	Smoldering	M	35	4		23.30	Present	-	-	-	-
10B ^β^	Chronic		38	7.9	23^#^	30.04	Present	-	-	-	-
11	Chronic	F	73	1.5	132^#^	3.71	Present	-	-	-	-
12	Chronic	M	35	4.5	38	22.70	Present	NA	LOH	NA	LOH
13	Chronic	M	20	30.7	84^#^	25.90	Absent	-	-	-	-
14*	Chronic	F	72	4.9	96^#^	29.67	Present	-	-	-	-
15 ^β^	Chronic	M	40	4.7	10	86.79	Present	-	-	MSI	MSI
16	Chronic	F	51	11.3	13	117.23	Present	NA	-	-	-
17*	Chronic	F	48	5.2	36^#^	8.48	Present	-	-	-	-
18	Chronic	M	42	4.4	6	27.76	Absent	-	-	-	-
19A ^β^	Chronic	M	32	9.5		22.14	Absent	-	-	-	-
19B ^β^	Acute		32	93.3	3	99.02	Absent	-	-	-	-
20	Acute	M	35	65.3	1,5	102.55	Present	NA	-	NA	-
21	Acute	F	39	26.9	4	37.00	Absent	-	-	-	-
22	Acute	F	73	16.3	1	40.25	Present	-	-	-	-
23	Acute	F	64	36.2	12	38.38	Present	-	LOH	-	-
24	Acute	F	19	306.0	0,5	92.72	Present	-	-	-	-
25	Lymphoma	M	63	1.2	9	6.02	Absent	-	-	-	-

(*) ATL associated with HAM/TSP.

(^β^) Patients that presented ATL progression during follow up. A second sample collected after progression were included for patients 10 and 19. For patient 15, that progress from chronic to acute form of ATL, no sample of acute phase was available.

(^£^) This patient presented fluctuations in lymphocytosis with low values of LDH and without systemic involvement of the disease, so he was classified as smoldering type.

(^#^) Patients still alive.

Abbreviations: M, male; F, female; MSI, microsatellite instability; LOH, loss of heterozygosity; -, no microsatellite alteration; NA, normal DNA sample had not amplified.

### Ethics statement

This study was approved by the Ethics Committee of the Hospital Climério de Oliveira from the Federal University of Bahia (UFBA). Informed written consent was obtained from all study participants or their guardians on their behalf (patient #1 and #3).

## Results

**Figure 1:**
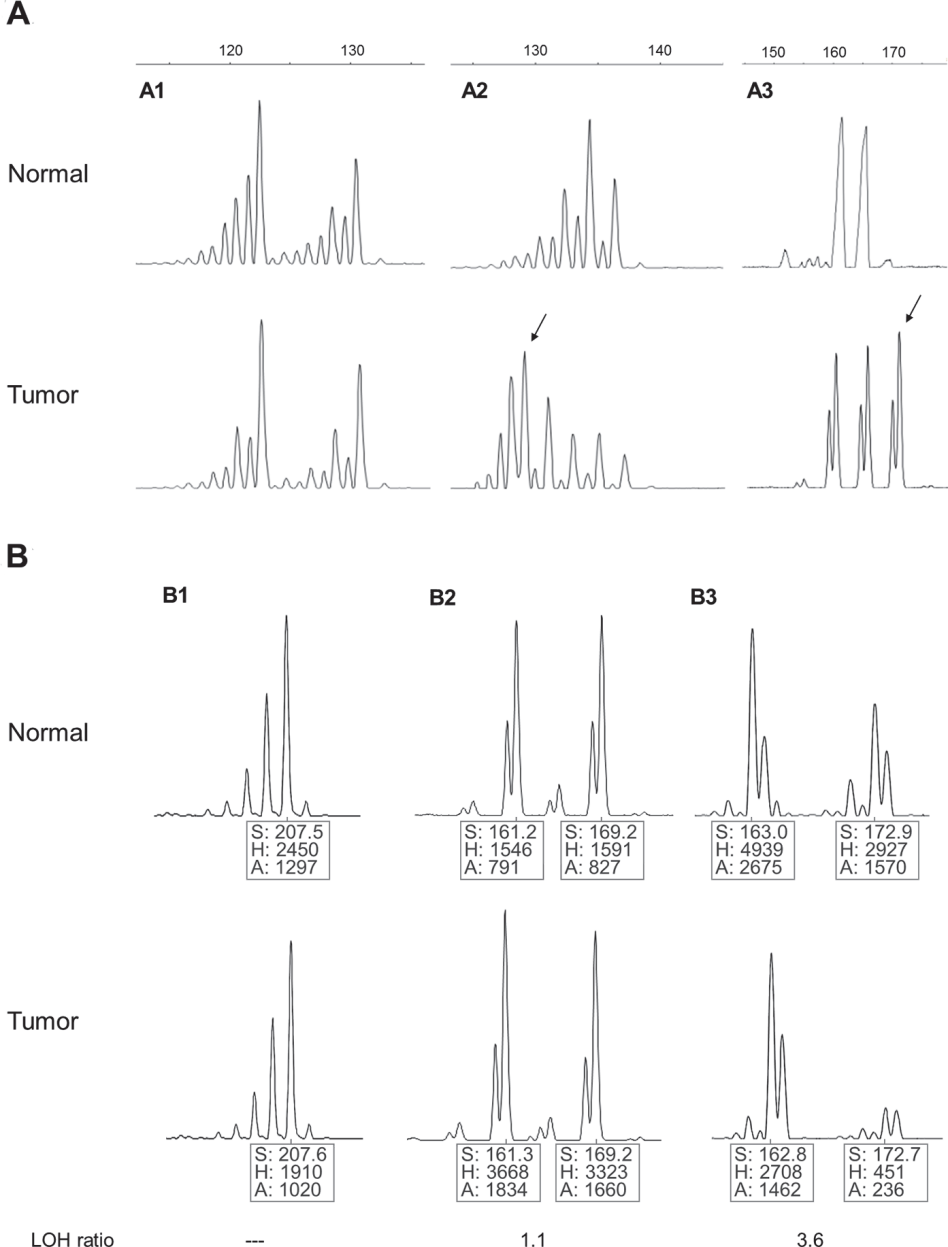
Assessment of microsatellite instability—MSI (a) and loss of heterozigosity—LOH (b) in patients with adult T cell leukemia-lymphoma (ATL). (a1) Tumor sample without allelic shift compared with normal sample (marker D10S191 – case 22). (a2) Patient showing allelic shift in tumor sample indicating the presence of microsatellite instability (D10S191 marker—case 8). (a3) Patient showing a novel allele in tumoral sample indicating the presence of microsatellite instability (marker D11S1391 – case 15). The arrows indicate allelic shift. (b1) The presence of a single peak in both normal and tumor DNA indicates a non informative case for LOH analysis (marker D10S190 – case 1). (b2) The presence of two peaks in normal and tumor DNA indicates an informative case for LOH analysis (marker D11S1391 – case 9). (b3) The decrease in peak height in one of two alleles in tumor DNA indicates allelic imbalance (LOH) (marker DCC – case 12); S, Size of PCR product (in pb); H, Fluorescence intensity of peak; A, area of peak.

MSI was found in four of the 24 patients evaluated (16.6%) (Table 1; Fig. 1A), three of which consisted of smoldering and one of chronic ATL. Two patients were males and two females. In the smoldering ATL, MSI was found in only one locus, in chromosome 10 in two patients (in marker D10S190 in patient #6 and in D10S191 in patient #8) and in chromosome 18 in the other.

However, in the chronic ATL (patient #15) MSI was present in two chromosomes, 11 and 18 (D11S1391 and DCC locus), being categorized as a replication error (RER+) phenotype. Median age at diagnosis was similar between the smoldering patients with and without MSI (52 and 56 years respectively). The chronic patient with MSI was 40 years old at diagnosis, similar to the median age at diagnosis of the chronic group without MSI, which was 45 years.

The three smoldering patients with MSI are still alive after more than four years of follow-up and the disease has not progressed to more aggressive types of ATL in any of them. Remarkably, one of these patients was diagnosed with ATL in adolescence in 1991 [17]. The chronic ATL with MSI in two loci progressed to acute ATL eight months after diagnosis. Unfortunately, an additional sample after progression was not available. This patient in the chronic phase presented a lymphocyte count of 4.7 ×10^9^ cells/L, lower than the median lymphocyte count of the whole chronic group (8.1 ×10^9^ cells/L). Patient #10 progressed from the smoldering to chronic ATL and patient #19 from the chronic to acute ATL. These two patients did not present MSI in the loci here considered. Interestingly, MSI was not detected in any of the five acute patients. These patients presented high lymphocyte counts (range of 16.3 to 306.0 × 10^9^ cells/L, Table 1) and poor survival, with a median survival time (MST) of 1.5 months.

LOH was found in four of the 24 patients (16.6%) (Table 1; Fig. 1B), and was detected in only one locus in three patients, two of the smoldering and one of the acute type, while in the fourth patient, a chronic type, LOH was present in two loci. In all four patients, LOH was detected in chromosome 10 (the smoldering ATL #5 at D10S190 and the other patients at D10S191). Additionally, LOH in the chronic patient was also detected in chromosome 18. Levels of lymphocyte counts in the chronic and acute patients who presented LOH were at similar range to the median of the respective clinical type group. None of the chronic or smoldering patients with LOH progressed to aggressive types of ATL. However, in patients with progression, LOH was not observed.

In only one patient, MSI and LOH were observed simultaneously. This patient presented the smoldering type of ATL. MSI was detected in chromosome 10 while LOH in chromosome 18.

Eight of the 24 patients also had HAM/TSP, six with the smoldering and two with the chronic type. Microsatellite alterations were present in three of the smoldering patients (Table 1) whereas the chronic patients did not show LOH in any loci examined.

## Discussion

The frequency of MSI in ATL patients in the current study was lower compared to previous reports [12],[13]. This may be partially due to the few acute and lymphoma ATL patients who were included. However, the absence of MSI in six acute patients was unexpected. Interestingly, we also found MSI in the loci previously reported in ATL patients from Japan [12], another endemic area for ATL.

To the best of our knowledge, this is the first report of MSI detection in less aggressive forms of ATL. In Japanese studies, the presence of MSI has only been reported in the acute and lymphoma types of ATL and the authors associated these alterations with progression of disease [12],[13]. In other hematological malignancies, the presence of MSI has also been linked to disease progression [18],[19]. The present results suggest that the deficiency of the DNA mismatch repair system may also constitute a mechanism in the development of smoldering and chronic ATL, not only being related to the progression of ATL to more aggressive forms, but also to the development of ATL itself. Our results suggest that the presence of MSI is not necessarily an indicator of poor prognosis in ATL as only the chronic patient with MSI progressed to acute ATL while the smoldering patients did not progress and are still alive after several years of follow-up. Since it was not possible to obtain a sample from the chronic patient #15 in the acute phase, any additional assessment of MSI after progression of the disease was precluded. The progression observed in two other patients without MSI in the loci included here may be related to alterations in other loci, or to other genetic or epigenetic alterations.

The frequency of LOH detected in our study was also lower than expected. Hatta et *al*. [14] evaluated 39 chromosome arms in patients with acute and lymphoma ATL and they reported a high frequency of LOH (91%). This discrepancy is probably related to the different loci and clinical types evaluated. It is noteworthy that LOH was present in the D10S191 locus in three different clinical types: the acute, chronic and smoldering types. As observed in another study, the presence of LOH in the D10S191 locus has also been associated with both initial and advanced stages of colorectal cancer [20].

Microsatellite alterations were observed in 50% of the smoldering patients with HAM/TSP. In Bahia, 14% of the ATL patients were shown to have HAM/TSP, which is more frequent among the smoldering patients [21]. In all these patients, HAM/TSP was diagnosed prior to ATL. There is no evidence in the literature that HAM/TSP could constitute a predisposing factor for ATL. HAM/TSP is characterized by high proviral load. As reported by Gillet et al [22], in HAM/TSP patients elevated level of proviral load is related mainly to the increase of the number of different clones more than to the relative abundance of these clones. It may be possible that the increase of infected clones in HAM/TSP patients in relation to asymptomatic carriers may favor the appearance of a new clone with oncogenic capacity conferred by the occurrence of microsatellite instability. Other mechanisms could also be implicated.

The number of patients included in the current study is too small to infer any relation between the presence of MSI or LOH in the loci examined and the earlier development of ATL. Even if patient #1 developed smoldering ATL very early [17], the median age at diagnosis was similar between the smoldering patients with microsatellite alterations and the patients without these alterations. The absence of a relationship between microsatellite alterations in the studied loci and lymphocytosis or prognosis, indicates that in these patients other loci or genetic or epigenetic alterations may be related to cell proliferation and prognosis.

MSI and LOH were detected even in the presence of a small percentile of infected cells in the sample (around 9% in smoldering patients that presented the lower median proviral load value between clinical types). In this sense, in patient #5, who showed the lowest proviral load with 5% of infected cells LOH was detected in locus D10S190.

The present findings show that MSI and LOH may also be associated with the less aggressive ATL and not only with the aggressive types [11]–[13]. Moreover, microsatellite alterations may occur simultaneously in different chromosomes in the smoldering and chronic types, as previously reported in aggressive forms of ATL [12]–[14]. These results suggest that in the less aggressive types of ATL, there may be alterations in the DNA mismatch repair system [11]. LOH represents somatic deletion in tumor DNA and may be related to inactivation of a tumor suppressor gene [23]. Moreover, possible tumor suppressor genes located in these regions could be inactivated in the less aggressive forms. The presence of microsatellite alterations in cases with the smoldering and chronic types constitutes a new finding in ATL oncogenesis.

## Supporting Information

S1 Checklist STROBE Checklist. (DOC)

## References

[1] Uchiyama T, Yodoi J, Sagawa K, Takatsuki K, Uchino H. (1977) Adult T-cell leukemia:clinical and hematologic features of 16 cases. Blood 50: 481–492. 301762

[2] Gessain A, Cassar O. (2012) Epidemiological aspects and world distribution of HTLV-1 infection. Frontiers in Microbiolgy 3: 388 10.3389/fmicb.2012.00388 23162541 PMC3498738

[3] Yamaguchi K, Watanabe T. (2002) Human T lymphotropic virus type-I and adult T-cell leukemia in Japan. Int J Hematol 76: 240–245. 10.1007/BF03165123 12430931

[4] Murphy EL, Hanchard B, Figueroa JP, Gibbs WN, Lofters WS, et al. (1989) Modelling the risk of adult T-cell leukemia/lymphoma in persons infected with human T-lymphotropic virus type I. Int J Cancer 43: 250–253. 10.1002/ijc.2910430214 2917802

[5] Amano M, Kurokawa M, Ogata K, Itoh H, Kataoka H et al. (2008) New entity, definition and diagnostic criteria of cutaneous adult T-cell leukemia/lymphoma: human T-lymphotropic virus type 1 proviral DNA load can distinguish between cutaneous and smoldering types. J Dermatol 35:270–275. 10.1111/j.1346-8138.2008.00465.x 18477226

[6] Bittencourt AL, da Gracas Vieira M, Brites CR, Farre L, Barbosa HS (2007) Adult T-cell leukemia/lymphoma in Bahia, Brazil: analysis of prognostic factors in a group of 70 patients. Am J Clin Pathol 128: 875–882. 10.1309/2YGD1P0QCVCWBLDX 17951212

[7] Gessain A, Barin F, Vernant JC, Gout O, Maurs L, et al. (1985) Antibodies to human T-lymphotropic virus type-I in patients with tropical spastic paraparesis. Lancet 2:407–410. 10.1016/S0140-6736(85)92734-5 2863442

[8] Shimoyama M (1991) Diagnostic criteria and classification of clinical subtypes of adult T-cell leukaemia-lymphoma. A report from the Lymphoma Study Group (1984–87). Br J Haematol 79: 428–437. 10.1111/j.1365-2141.1991.tb08051.x 1751370

[9] Matsuoka M, Jeang KT (2007). Human T-cell leukaemia virus type 1 (HTLV-1) infectivity and cellular transformation. Nat Rev Cancer 7:270–80. 10.1038/nrc2111 17384582

[10] Zhu YM, Das-Gupta EP, Russell N (1999) Microsatellite instability and p53 mutations are associated with abnormal expression of the MSH2 gene in adult acute leukemia. Blood 94: 733–740. 10397740

[11] Offman J, Opelz G, Doehler B, Cummins D, Halil O, et al. (2004) Defective DNA mismatch repair in acute myeloid leukemia/myelodysplastic syndrome after organ transplantation. Blood 104: 822–888. 10.1182/blood-2003-11-3938 15090454

[12] Hatta Y, Yamada Y, Tomonaga M, Miyoshi I, Said JW, et al. (1998) Microsatellite instability in adult T-cell leukaemia. Br J Haematol 101: 341–344. 10.1046/j.1365-2141.1998.00710.x 9609532

[13] Hayami Y, Komatsu H, Iida S, Utsunomiya A, Hanada S, et al. (1999) Microsatellite instability as a potential marker for poor prognosis in adult T cell leukemia/lymphoma. Leuk Lymphoma 32: 345–349. 10037032 10.3109/10428199909167395

[14] Hatta Y, Yamada Y, Tomonaga M, Said JW, Miyosi I, et al. (1998) Allelotype analysis of adult T-cell leukemia. Blood 92: 2113–2117. 9731069

[15] Hatta Y, Yamada Y, Tomonaga M, Miyoshi I, Said JW, et al. (1999) Detailed deletion mapping of the long arm of chromosome 6 in adult T-cell leukemia. Blood 93: 613–616. 9885223

[16] Canzian F, Salovaara R, Hemminki A, Kristo P, Chadwick RB, et al. (1996) Semiautomated assessment of loss of heterozygosity and replication error in tumors. Cancer Res 56: 3331–3337. 8764130

[17] Bittencourt AL, Barbosa HS, Requiao C, da Silva AC, Vandamme AM, et al. (2007) Adult T-cell leukemia/lymphoma with a mixed CD4+ and CD8+ phenotype and indolent course. J Clin Oncol 25: 2480–2482. 10.1200/JCO.2007.11.3043 17557960

[18] Tasaka T, Lee S, Spira S, Takeuchi S, Nagai M, et al. (1997) Microsatellite instability during the progression of acute myelocytic leukaemia. Br J Haematol 98: 219–221. 10.1046/j.1365-2141.1997.1672985.x 9233589

[19] Wada C, Shionoya S, Fujino Y, Tokuhiro H, Akahoshi T, et al. (1994) Genomic instability of microsatellite repeats and its association with the evolution of chronic myelogenous leukemia. Blood 83: 3449–3456. 8204873

[20] Shima H, Hiyama T, Tanaka S, Ito M, Kitadai Y, et al. (2005) Loss of heterozygosity on chromosome 10p14-p15 in colorectal carcinoma. Pathobiology 72: 220–224. 10.1159/000086792 16127298

[21] Bittencourt AL, Barbosa HS, Vieira MD, Farre L (2009) Adult T-cell leukemia/lymphoma (ATL) presenting in the skin: clinical, histological and immunohistochemical features of 52 cases. Acta Oncol 48: 598–604. 10.1080/02841860802657235 19165640

[22] Gillet NA, Malani N, Melamed A, Gormley N, Carter R, et al. (2011) The host genomic environment of the provirus determines the abundance of HTLV-1-infected T-cell clones. Blood 117: 3113–3122. 10.1182/blood-2010-10-312926 21228324 PMC3062313

[23] Fearon ER, Cho KR, Nigro JM, Kern SE, Simons JW, et al. (1990) Identification of a chromosome 18q gene that is altered in colorectal cancers. Science 247: 49–56. 10.1126/science.2294591 2294591

